# Hospitals and the Budget

**Published:** 1909-07-03

**Authors:** 


### HOSPITALS AND THE BUDGET.

A memorial signed by the chairman and treasurer of several of the most important institutions, representing amongst others the following hospitals, was forwarded to the Chancellor of the Exchequer on June 25th :—Cancer, Charing Cross, General Hospital Birmingham, General Hospital Bristol, General Infirmary Leeds, Great Northern Central, Guy's, Hospital for Sick Children, Hospital for Consumption Brompton, King's College, King Edward's Hospital Fund, London, Meath Hospital, Middlesex, Poplar, Queen Charlotte's, Queen's Hospital for Children, Royal Free, Royal Infirmary Liverpool, Royal Infirmary Manchester, Royal Infirmary Sheffield, Royal National Hospital for Consumption Ventnor, Royal London Ophthalmic, Royal Victoria Hospital Belfast, St. Luke's Hospital for Lunatics, St. Mary's, St. Thomas', Seamen's, University College, Victoria Hospital for Children, Westminster. The memorial is also supported and signed by representatives of a number of the greater charities of various kinds.

The text of the memorial is as follows :

"As the representatives of many of the largest and most important hospitals and charitable institutions in the kingdom, we desire to bring to your notice the effect which certain of the Budget proposals will have upon the incomes of these and similar charities.

"We submit that these institutions, in carrying on the work for which they have been organised, are, in a large measure, aiding the State by protecting, maintaining, and educating orphan, destitute, and feeble-minded children; by saving life; by nursing the sick, and fighting against disease; by caring for the afflicted; by providing for the necessities of our soldiers and sailors, their widows, and orphans; by succouring mariners shipwrecked upon our shores; by paying annuities to thousands who would otherwise be in the workhouse; by creating a more civilising public opinion; and by materially assisting in carrying out the laws of the land.

"Work so widespread and affecting the welfare of so many of his Majesty's subjects has, we feel, special claims upon the consideration of his Majesty's Ministers, and we are assured of your attentive interest on its behalf.

"In the first place we beg to point out that a large proportion of the revenue of all important charities is derived from legacies, and that it is becoming an increasing practice of testators to leave either the whole or a part of their residuary estates for charitable purposes. It is the experience of some of the institutions represented that of all bequests they receive, from 15 per cent. to 25 per cent. are from residuary estates; and, with our intimate knowledge of the finances of these hospitals and societies, we view with alarm the diminished receipts which must result in consequence of the increased estate, succession, and legacy duties which it is proposed to levy, and, in a lesser degree, as a result of the newly suggested method of valuing agricultural land for estate duty.

"All these additional duties will, in so far as they are payable out of the estates, proportionately reduce the residues received by the charities to which they have been bequeathed. The annual loss to them will amount to many thousands of pounds, which must necesarily mean a curtailment of the work they carry on.

"The seriousness of the position is aggravated by the fact that of late years charities have found it very difficult to raise sufficient funds to meet the growing demands made upon them. Increased taxation had already been urged by many contributors as a reason for the discontinuance or reduction of their gifts.

"We would earnestly ask that you will seriously consider whether the Finance Bill cannot be so amended as to grant exemption from legacy duty to bequests which, in the opinion of the commissioners, are for charitable purposes, in order that these institutions may be compensated for the loss of income which they will suffer in other directions.

"We would respectfully suggest that this exemption might be conveniently granted by a method similar to that by which charities now claim the repayment of income tax deducted from dividends upon their investments. This would secure a minimum loss to the national revenue, and would give to the commissioners discretionary powers as to the classes of charities entitled to exemption.

"Second, we further desire to ask that Section 25 of the Bill may also be amended so as to exclude from increment value duty land sold or let by charities for their benefit in any sense whatever. In this connection we would point out that the section as at present drafted would very seriously affect many institutions, such as hospitals, upon removal of their premises from one site to another.

"In conclusion may we urge that, inasmuch as the claims of charities to preferential treatment have already been recognised with regard to certain of the new financial provisions, it is not inconsistent to ask for these additional concessions that charitable institutions, which are admittedly doing great and beneficent work for the nation, may not be hampered by such severe losses as the present proposals of the Finance Bill would entail upon them."

